# A modified direct lateral approach for neck-preserving total hip arthroplasty: tips and technical notes

**DOI:** 10.1007/s10195-013-0224-4

**Published:** 2013-03-08

**Authors:** F. Pipino, M. Cimmino, A. Palermo

**Affiliations:** Policlinico di Monza, Via Amati 111, 20052 Monza, Italy

**Keywords:** Tissue-sparing surgery, Direct lateral approach, Preservation of the gluteus medius, Transverse ligament, Hip prosthesis, Preservation of the neck of the femur, Collum femoris preserving (CFP) system, Minimally invasive surgery

## Abstract

Tissue-sparing surgery for hip replacement aims to minimize muscle damage and conserve the femoral neck through the use of mini-prostheses. We propose a modification of the classical direct lateral access procedure that preserves the gluteus medius. Further advantages during the surgical phase include limited blood loss, visualization of the entire acetabulum, and sparing of the transverse ligament. Precise implantation is facilitated and normal biomechanics are preserved. The gluteus medius is divided longitudinally between the anterior third and posterior two-thirds to provide access to the gluteus minimus, which is detached from the femoral insertion together with a small portion of the vastus lateralis, forming a flap that exposes the underlying articular capsule. When the femoral head is revealed, a decision is made to either continue with its dislocation directly or to resect it and remove it separately to avoid damaging the gluteus medius during dislocation. Upon removal of the femoral head, with the limb flexed and slightly over-rotated, the acetabulum is completely visible. Limb length is maintained through the use of reference stitches on the gluteus minimus tendon and the proximal insertion of the vastus lateralis. In keeping with the minimally invasive philosophy, only pathological tissue is removed (marginal osteophytes, geodes, joint capsule, cartilage to the point of bleeding and pulvinar). We have performed more than 2,000 implants with this procedure since 1990. Advantages and potential critical points are discussed.

## Introduction

Tissue-sparing surgery (TSS) is the “philosophy” of respecting soft tissue and bone whenever possible. We believe that obtaining good functional and clinical results in prosthetic hip surgery requires that a balance be struck between the need for correct implant positioning and the need to respect tissues. For this reason, we proposed a modification of the classical direct lateral access procedure that can be used with the patient lying in the supine or lateral position [1]. Our modification preserves the anatomical integrity of the gluteus medius, which facilitates “step-by-step” visualization of the surgical anatomy; we have used it to implant more than 2,000 hip prostheses since 1990.

Advantages during the surgical phase are limited blood loss, preservation of the gluteus medius, complete visualization of the acetabulum, and sparing of the transverse ligament. Furthermore, it allows complete and precise removal of osteophytes, abrasion of the modulated acetabulum, and femoral neck preservation when a collum femoris preserving (CFP) prosthesis is chosen.

The procedure that we describe spares soft tissue and bone in line with TSS criteria and allows precise prosthesis implantation that preserves normal biomechanics. We discuss its advantages and potential critical points.

## Tissue-sparing surgery

Given the continual evolution of implants and surgical techniques, an increasing number of patients are requesting “performance” prostheses that allow early functional recovery, simplified re-operation, and greater attention to aesthetic needs. Minimally invasive prosthetic surgery achieves these objectives by combining conservative implants, reliable bearings, and increasingly small incisions.

But does MIS access consistently lead to successful implantation? There is no documented evidence comparing the relative efficacy of MIS and traditional accesses in terms of biological respect for tissues [2]. Although a study by Chung [3] appears to favor mini-incision accesses, it was actually based on incisions with a mean length of 9.2 cm. Another study [4] found no significant differences in rehabilitation between patients undergoing traditional lateral or mini-lateral access procedures.

There is also no documented benefit of the two-incision route. On the contrary, a randomized cadaver trial revealed that significantly more muscle mass is damaged by the passage of the rasps and stem during preparation and implantation with the two-incision method versus the mini-posterior technique (gluteus medius, 15.4 vs. 4.7 %, *p* = 0.0046; gluteus minimus 17.37 vs. 8.62 %, *p* = 0.002) [5]. A prospective randomized trial comparing the two-incision and mini-incision posterior procedures did not reveal differences in perioperative outcomes between these two approaches [6]. Alecci et al. [7] compared intra- and perioperative outcomes in patients undergoing surgery with the minimally invasive direct anterior approach or the standard lateral approach, and reported that the minimally invasive approach provided better perioperative outcomes. The mini-anterior or Smith–Petersen approach is certainly the most anatomical, although it sacrifices a branch of the anterior circumflex artery and exposes the lateral cutaneous femoral nerve to risk. It provides optimal access to the acetabulum, but it is difficult to implant the stem without damaging the gluteus medius and tensor muscles. Consequently, Berger proposed the two-incision approach (anterior for the acetabulum, and lateral for the femur) [8].

Tissue-sparing surgery is a surgical philosophy that reflects an attitude of the greatest respect for the person and, surgically, for the soft tissues and bone [9]. At the same time, it must allow safe, conservative, and correct implant positioning [10]. This is facilitated by an operative field that is clean, unobstructed, and suitable for positioning the prosthesis. The surgical technique only requires the removal of pathological hip tissue (cartilage, femoral head, osteophytes, and geodes) and allows the femoral neck to be preserved [11].

## Surgical technique

**Fig. 1:**
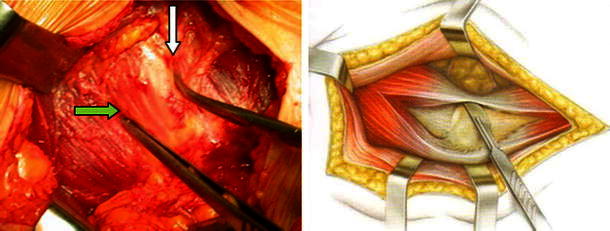
*Left*: photograph of the patient’s head showing the intramuscular septum between the anterior third and the posterior two-thirds of the gluteus medius (*green arrow*), and delimitation of the anterior border (*white arrow*). *Right*: drawing of the separation of the two parts of the muscle, with the formation of an anterior flap that includes the anterior third of the gluteus medius and the anterior half of the gluteus minimus, joined to the anterior portion of the tensor fasciae latae by the conjoint tendon, which is detached from the femur. The drawing is taken from the* Atlante di Chirurgia Ortopedica* (*Orthopedic Surgery Atlas*), edited by F. Pipino and published by Gerni Editore as a special edition (color figure online)

The preferred access for prosthetic hip TSS is a modified version of the direct lateral approach developed by McFarland, Bauer, and Hardinge [12–14]. The skin incision along the midline of the greater trochanter includes the fascia and is about 10 cm long. Hemostasis is easier, and further facilitated by the use of a Charnley retractor. The operative field is usually clear enough that the gluteus medius can be recognized. Unlike other accesses that sacrifice the fibers of the gluteus medius, the fibers of the anterior third are separated longitudinally from those of the posterior two-thirds [1] (Fig. 1).

**Fig. 2:**
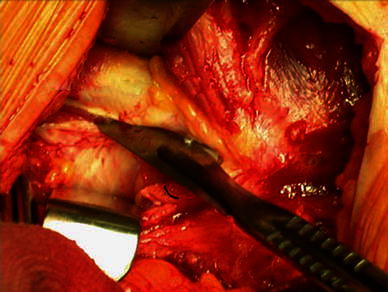
Sectioning and detachment of the gluteus minimus

**Fig. 3:**
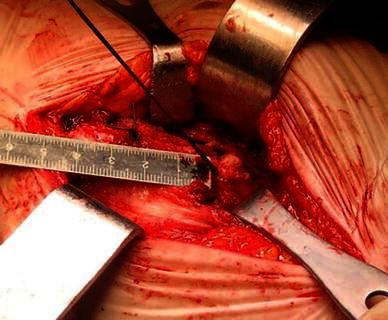
Measuring the length of the limb by means of two reference stitches applied to the gluteus minimus and vastus lateralis

The tendon of the gluteus minimus is recognizable by opening the fibers of the gluteus medius among its 2/3 posterior and 1/3 anterior. The tendon of the gluteus minimus is then dissected longitudinally in half (Fig. 2). The anterior half is elevated from the femur together with a small portion of the vastus lateralis, thus showing the capsule. The posterior half is detached from the greater trochanter after a stitch is positioned. A second stitch is positioned on the vastus, and their distance is measured for length evaluation, since the gluteus minimus tendon is not extensible (Fig. 3).

The articular capsule is then carefully isolated and removed, revealing the head and neck. At this point a decision is made to either continue dislocation of the head or to resect it to avoid damaging the gluteus medius during dislocation.

**Fig. 4:**
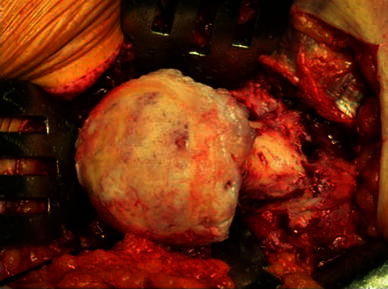
Dislocation of the femoral head

In the first case, the head can be easily dislocated with slight over-rotation and forced adduction of the limb (Fig. 4) and then placed into a sterile pocket arranged during preparation of the field. If instead the osteotomy is performed in two stages to avoid damaging the medioposterior part of the gluteus medius, then the head is removed subsequently using an appropriate instrument.

Removal of perimetral osteophytes clearly exposes the neck, thus making osteotomy possible at the isthmus or base, depending on the type of prosthesis chosen.

**Fig. 5:**
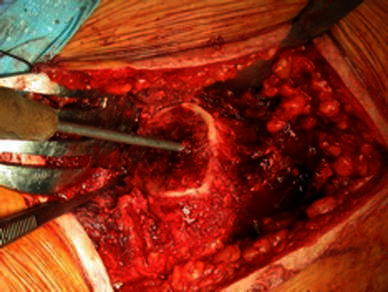
Locating the center of the femoral neck. The cylinder of the neck is filled with spongy bone that is mechanically suitable for the three-dimensional (especially rotatory) stabilization of neck-preserving prostheses [9, 10]

With the patient in a lateral position, moderate extrarotation is sufficient to reveal the femoral head and neck (Fig. 5).

**Fig. 6:**
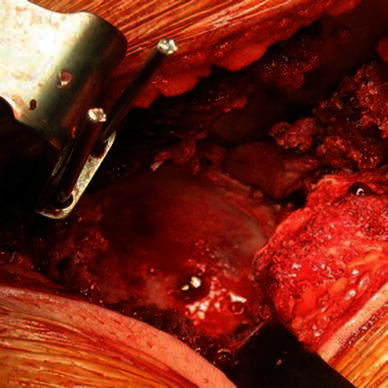
Exposure of the acetabulum and retraction of the femoral neck (if it is to be preserved) using the Homann lever supported by the posterior wall

Upon the removal of the femoral head, with the limb flexed and slightly over-rotated, the acetabulum is completely visible. It is exposed using two Homann levers (one anterior and one posterior), and a special retractor applied to the upper cotyloid rim (Fig. 6).

**Fig. 7:**
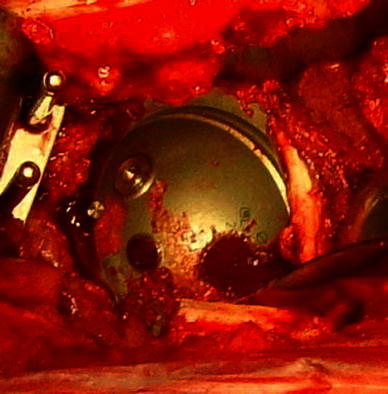
Preservation of the transverse ligament (clearly visible on the* right* at the inferior pole of the cup). This structure is a very useful reference point for determining acetabular version and may participate in modulating the elastic deformation of the bony acetabulum

In keeping with the philosophy of TSS, only pathological tissue is removed (marginal osteophytes, geodes, capsule, cartilage to the point of bleeding, pulvinar). The transverse ligament is left intact because it may be important for modulating elastic deformation of the bony acetabulum, although this is not documented in the literature. Moreover, practical experience has shown that it can guide orientation of the acetabular component of the prosthesis in anteversion (Fig. 7). In 2006, Archbold et al. [16] demonstrated its importance as a physiological guide for the orientation of this component in a study of 1,000 cases.

Particular care must be taken to avoid damage to the robust psoas tendon, which passes behind—and is in contact with—the inferomedial margin of the acetabulum, and may be damaged during the preparation or implantation of the cup. In this regard, there is debate over whether the medial capsulotomy should be performed from the inside. This is easy to perform using our procedure (Fig. 3), which exposes the entire acetabulum and increases postoperative limb abduction (especially when the capsule is retracted and fibrous). However, capsulotomy removes a natural barrier between the psoas tendon and the inferior spur and stem collar (if present), particularly when a relatively large part of the medial capsule is removed. When using a T.O.P. cup (Waldemar Link, Germany), the lower border is removed to avoid any impingement on the psoas, which can cause characteristic persistent inguinal pain.

When the patient is lying in a lateral position there is better exposure of the femoral neck than in the supine position, the lateral position allows complete visualization of the angle of declination, as the axis of the femur can be observed all the way to the condyles.

### Advantages and disadvantages

Table 1 lists the main advantages and disadvantages of various phases of the surgical procedure and compares them to the alternative (anterior or posterolateral) methods.

**Table 1:** Fibers sparing of medius gluteus muscle, between the posterior two-thirds and one-third on front

Surgical phase	Advantages	Disadvantages
“Longitudinal” skin incision	Good exposure: it can be extended proximally and distally as required	Less scarring might be obtained with an “oblique” incision
Incision of the subcutaneous tissue and fascia without separation	Less bleeding (especially with the timely use of a Charnley retractor)	The fascia is less visible when incising and suturing, as it is not exposed by separation
Exposure and splitting of the gluteus medius between the anterior and middle thirds	Extensive preservation of the split gluteus medius, whose anterior third is retracted anteriorly together with the anterior half of the gluteus minimus and the anterior half of the vastus lateralis	The presence of the anterior branch of the superior gluteal nerve about 4–5 cm from the apex of the greater trochanter makes it difficult to extend the deep field proximally
Exposure of the aponeurosis of the gluteus minimus and its longitudinal incision in half to the apex of the greater trochanter. Elevation of the anterior flap and application of reference stitches to evaluate limb length before and after implantation	This allows its anterior half to be moved to form the anterior flap together with the anterior third of the gluteus medius. Greater respect for the anterior third of the gluteus medius, and better exposure of the capsule. The posterior half, separated from the capsule and transected, is used to monitor limb length with two reference stitches (one on the gluteus minimus tendon, another on the vastus lateralis)	The difficulty involved in detaching the conjoint tendon, with the possibility that the anterior flap will be divided into two parts. The need to coagulate the vascular network near the vastus
Capsular phase. Separation with exposure of the anterolateral wall. Capsulectomy. Osteophytectomy	Facilitates broad and precise anterolateral capsulectomy. View of the femoral neck and axis, with the possibility of either two-stage neck osteotomy or dislocation (the usual practice). The removal of anterolateral osteophyes and limbus (even if calcified)	It does not expose the medial wall of the capsule, which is only resected subsequently
Dislocation of the head. Osteotomy of the neck. Osteophyte removal	Optimal freeing to the base of the neck. Possibility of removing the osteophytes of the head and neck, which is necessary for correct identification of the isthmus (1.5 cm from the greater trochanter)	The passage of the head may damage the posterior part of the gluteus medius. Limited detachment of its trochanteric insertion or two-stage osteotomy (in the case of particularly large and even sub-ankylosed heads) may be preferable
Exposure of the acetabulum and medial capsulotomy	The medial capsule is clearly visible. Separation and sectioning or removal are possible, even when adherent. Optimal visualization of the acetabulum and a greater range of motion, which is particularly useful for postoperative recovery of abduction. The psoas tendon and its relationship with the prosthesis (cup or collar of the stem) are visible	Medial capsulectomy removes a protective barrier (the capsular wall normally shields the psoas) and favors impingement on the psoas, which can lead to persistent medial inguinal pain
Preparation of the acetabulum	Complete removal of osteophytes, even if medial or at the bottom. Removal of the pulvinar (even if covered by an ossified roof). Exact depth of rasping to the point of eliminating the pulvinar from the fossa. Exposure of the transverse ligament, which is respected as part of the biodynamics of the acetabulum and as a guide for the correct anteversion of the cup	Risk of lateralizing the center of rotation because of insufficient cup depth
Implantation of the cup	The access also facilitates orientation. In the case of a T.O.P. cup, the insert can be rotated posteriorly to form an antiluxation long posterior wall because of its two equators	Preserving the neck of the femur is more difficult. The neck needs to be displaced backwards, and this is partially obstructed by the psoas (this does not occur with the posterior route because the neck is displaced forward and holds the psoas)
Implantation of the stem	Greatly facilitated without sacrificing the gluteus medius or other structures	Need to reveal the greater trochanter in the case of straight stems
Reduction and evaluation of the length of the limb before and after implantation	The reference stitches on the gluteus minimus and vastus lateralis are useful. The distance between the two stitches is measured with the limb in repose (neutral)	The lateral body position complicates this
Closure in layers	(a) Attention when reinserting the conjoint tendon together with the anterior flap (b) Suture gluteus medius	Some difficulty in identifying the conjoint tendon, especially if it is accidentally broken or labile

In conclusion, the innovation of the proposed route over the classical lateral approaches of Hardinge and Bauer is its greater preservation of the gluteus medius and the fact that the gluteus minimus is used to gain access to the capsule and to calculate limb length.

## Final considerations

Our detailed description of the proposed access route and, particularly, the analysis of its advantages and disadvantages in comparison with the classic lateral transgluteal route show that it is particularly useful in the context of TSS. This is the rationale underlying our technique, which aims to spare bone and soft tissues while optimizing hip biomechanics, through the use of mini-prostheses [10, 11, 15]. To ensure that we obtain the optimal biomechanics, we should not be induced into making “blind” interventions that are less invasive, employ incisions that are too small, or use surgical approaches that do not allow anatomical structures to be viewed as they are successively reached.
